# Beneficial Effects of *Limosilactobacillus reuteri* PBS072 and *Bifidobacterium breve* BB077 on Mood Imbalance, Self-Confidence, and Breastfeeding in Women during the First Trimester Postpartum

**DOI:** 10.3390/nu15163513

**Published:** 2023-08-09

**Authors:** Franco Vicariotto, Patrizia Malfa, Michela Torricelli, Lisa Lungaro, Giacomo Caio, Vincenzo De Leo

**Affiliations:** 1 Humanitas-San Pio X Hospital, 20159 Milano, MI, Italy; 2 Synbalance Srl, 21040 Origgio, VA, Italy; 3 Obstetrics and Gynecology, Department of Pediatrics, Obstetrics and Reproductive Medicine, University of Siena, 53100 Siena, SI, Italy; michelatorricelli@libero.it; 4 Department of Translational Medicine, University of Ferrara, 44121 Ferrara, FE, Italy; lisa.lungaro@unife.it (L.L.); caigmp@unife.it (G.C.); 5 Mucosal Immunology and Biology Research Center, Massachusetts General Hospital-Harvard Medical School, Boston, MA 02114, USA; 6 Medical Policlinic Colledoro, 53100 Siena, SI, Italy; vincenzo.deleo@unisi.it

**Keywords:** gut–brain axis, postpartum, depression, *Limosilactobacillus reuteri* PBS072, *Bifidobacterium breve* BB077, delivery, breastfeeding, probiotics

## Abstract

**Background:** The post-delivery period could be characterized by psychological distress (e.g., anxiety, sadness, and irritability), leading to postpartum depression (PPD). **Objective**: The present clinical study assesses the effect of probiotic supplementation containing *Limosilactobacillus reuteri* PBS072 and *Bifidobacterium breve* BB077 (4 × 10^9^ CFU/day) on the mother’s mood and breastfeeding quality during the first trimester after delivery. **Methods**: A Randomized, Double-Blind, Controlled (RDBPC) trial was carried out on 200 healthy new mothers divided into an active group taking a supplement containing *Limosilactobacillus reuteri* PBS072 and *Bifidobacterium breve* BB077 (4 × 10^9^ CFU/day) plus multivitamins and a control group (multivitamin complex only) for 90 days. Symptoms related to maternal depression and breastfeeding quality were evaluated at days 45 and 90 using the Edinburgh Postnatal Depression Scale (EPDS) and the Breastfeeding Self-Efficacy Scale—Short Form (BSES-SF). **Results**: At days 45 and 90, the probiotic treatment significantly ameliorated the mothers’ mood compared to the control treatment (*p* < 0.001). Likewise, the breastfeeding quality and the baby’s cries significantly improved in the probiotic group (*p* < 0.001). **Conclusions**: Microbiota alterations could influence a post-delivery woman’s mental state. According to our results, *L. reuteri* PBS072 and *B. breve* BB077 are potential candidates that are able to improve stress resilience in the postpartum period.

## 1. Introduction

Postpartum depression (PPD) is a subtype of a depressive disorder affecting a large number of women immediately after childbirth, which could seriously compromise both the mother’s and the newborn’s health [1,2]. 

Around 60–80% of new mothers experience “baby blues” a few days after delivery, a condition characterized by feelings of sadness, loneliness, worthlessness, restlessness, and anxiety, which usually resolve within the first two weeks [3,4]. This temporary discomfort, characterized by unstable mood and crying crises, is caused by the rapid hormonal and psychic adaptation processes happening during the first weeks after childbirth. Sometimes, these feelings persist for a longer period, leading to psychic fragility, stress, and anxiety [5]. PPD is a long-lasting depressive disorder that occurs 4–6 weeks after childbirth in which women experience depressed mood, loss of interest and pleasure, loss of confidence and self-esteem, excessive guilt, difficulty concentrating, and sleep and appetite disturbances [6]. The prevalence of PPD is around 10–20%, although it varies according to the different cultures and income levels of the studied countries [7].

Generally, women are subjected to many biological variations after delivery, such as altered levels of sexual hormones (oestrogen and progesterone), lactogenic hormones (oxytocin and prolactin), and increased amount of stress hormones (e.g., cortisol), correlated with a dysregulation in the hypothalamic–pituitary–adrenal (HPA) axis [8]. However, these hormonal imbalances lead to the onset of depression only in women with a predisposition for mood disorders [9]. Other factors playing a role in physiological changes after childbirth are neurotransmission dysfunction (i.e., γ-aminobutyric acid and glutamate) and monoamines (i.e., serotonin and dopamine) [10]. Common changes in the mother’s normal daily routine, such as sleep disturbances and alteration in appetite, could lead to diurnal variations in mood, loss of concentration, and irritability, which altogether are a possible cause for PPD development [7].

Another recurrent feeling that women might experience in the first trimester after delivery is a sense of guilt, arising from the difficulty in looking after their baby. Even though this emotion has a serious impact on both the mother’s and the child’s lives, it is very often underestimated, and it is not reported by women because of the fear of being labelled as “bad mothers” [11].

The first six months after birth have a pivotal role in a child’s physical and psychological development. For this reason, PPD can have serious consequences for the baby. Uncertain affection and reduced psychomotor and cognitive development are just some of the possible effects, which can globally affect the infant’s development, especially in terms of intelligence and language learning ability [1,12]. One of the most important leading consequences of women’s depressive mood after delivery is difficulty in lactation [13]. Breastfeeding is of paramount importance both for the newborn’s optimal immune system development and for the mother’s health, since it reduces type 2 diabetes, cardiovascular risk, and breast and ovarian cancer development [14,15]. Sometimes, lactation could cause mastitis, an inflammation of the breast tissue, usually followed by infection. It is a common and debilitating disease, which often presents on the cessation of exclusive breastfeeding. Mastitis and breast abscesses are usually associated with the pathogens *Staphylococcus aureus*, *Staphylococcus epidermidis*, and some *Corynebacterium* that are known to produce biofilms and show multidrug resistance. For these reasons, antibiotic treatment is frequently ineffective, causing, in many cases, breastfeeding cessation [16,17,18]. 

Nowadays, the current best practices for PPD involve pharmacological interventions, cognitive therapy, psychological support and, in the worst cases, hospitalization [19]. Thus, the development of non-invasive and non-harmful treatments to support new mothers’ health is eagerly required. 

In this scenario, probiotics have been extensively studied due to their ability to improve the gut microbiota (GM) and eubiosis. Indeed, such microorganisms affect the host’s health, influencing both the gastrointestinal and the immune system [20,21]. Bacteria living in the gut are also able to modulate brain functions through a bi-directional communication pathway, named the “gut–brain axis” [22]. Such a pathway includes neuroendocrine secretions, short-chain fatty acid production, and autonomic nervous system stimulation [23]. Consequently, intestinal dysbiosis can be associated with a wide range of psychological problems, including neuropsychiatric disorders, depression, and anxiety, that are characterized by increased levels of pro-inflammatory cytokines and oxidative stress [24,25]. Thus, an alteration in the intestinal microbial composition can affect the gut–brain communication, leading to the onset of depressive disorders [26]. 

*Bifidobacterium* strains are prevalent in the human gut microbiota and produce several metabolites (e.g., acetic and lactic acid) that are beneficial for intestinal barrier function and the immune system [27]. *Limosilactobacillus reuteri* is a probiotic with a high mucosal colonization capacity, a good gastrointestinal survival rate (as it usually is present in the human intestine), and recognized immunomodulatory properties. Also, *Limosilactobacillus reuteri* strains have antimicrobial features that depend on organic acid production (i.e., lactic, acetic, propionic, and phenyl lactic acids) and antibacterial molecules (i.e., hydrogen peroxide, carbon dioxide, ethanol, diacetyl, and acetaldehyde, bacteriocins, reuterin, and reutericyclin). These metabolites contribute to lowering the pH and inhibit the growth of pathogens [28].

In vitro studies suggest that *Limosilactobacillus reuteri* PBS072 and *Bifidobacterium breve* BB077 can influence the endogenous production of GABA and serotonin and improve stress-related parameters. Following these promising results, two clinical trials were carried out in different target populations, such as stressed students during an exam session and employees in the early pandemic, showing an improvement in cognitive functions, mood, and sleep quality [29,30].

The primary aim of the present study was to determine whether a probiotic supplement containing *Limosilactobacillus reuteri* PBS072 and *Bifidobacterium breve* BB077 could improve new mothers’ psychological and physical wellbeing by reducing the risk of developing PPD, through the modulation of the gut–brain axis. As a secondary outcome, self-confidence and breastfeeding ease were evaluated as a marker of the mothers’ mental state in the postpartum period. Indeed, these two outcomes are related to the lactation performance, decreasing excessive baby crying and reducing the production of intestinal gas (i.e., infant colic), due to a relaxed attitude of the new mothers.

## 2. Materials and Methods

### 2.1. Study Design, Population, and Products

A multicentre, randomized, double-blind, controlled clinical study was carried out in Italy, in compliance with the Helsinki Declaration (1964) and its amendment. This study’s protocol was approved by the “Independent Ethical Committee for Non-Pharmacological Clinical Studies” (Genova, Italy). Written informed consent was obtained from all subjects before enrolment. This study was registered at the ISRCTN registry (registration number: ISRCTN99047904). Subject enrolment was carried out between July 2020 and July 2021. 

**Figure 1:**
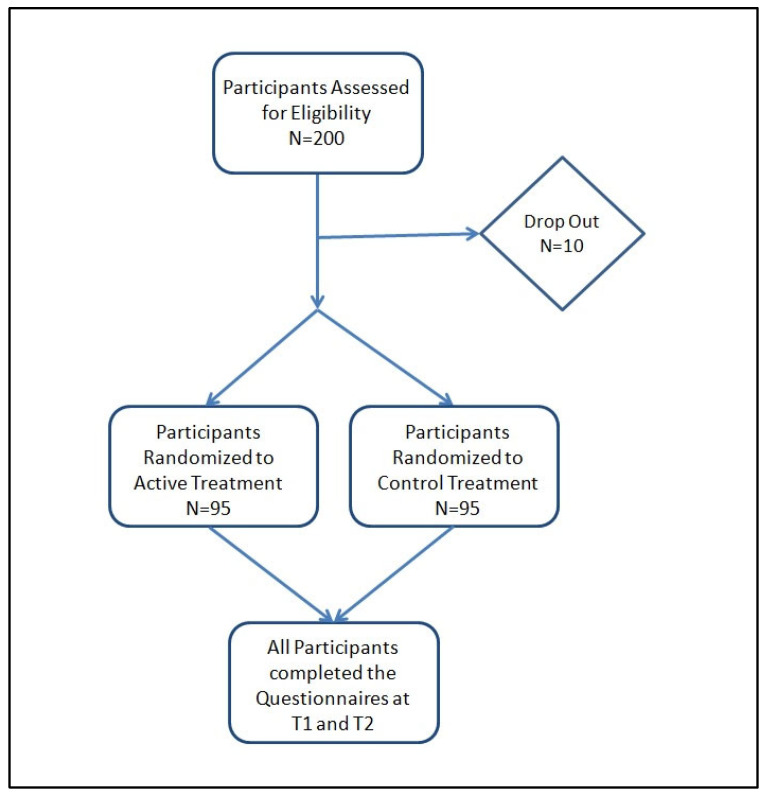
Flowchart of this study.

Two hundred healthy Italian (Caucasian) pregnant women, aged between 18 and 50 years old, were enrolled according to the inclusion and exclusion criteria reported in Table 1. Of these, 10 subjects dropped out; thus, a total of 190 subjects completed the treatment (95 for the active treatment and 95 for the control one). The average age of the women was 32.54 ± 4.16 years. This study started on the day of the first intake of the product (T0), within 3 (±1) days maximum after delivery. No adverse events were reported, and both products used in this clinical study were well tolerated. The level of compliance with the treatment was high. Figure 1 shows a visual overview of this study’s design (flowchart).

**Figure 2:**
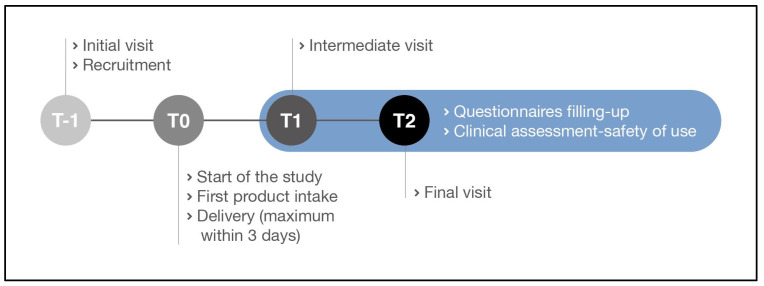
Study timeline.

Subjects were randomly assigned to 2 groups according to a randomization list previously generated using the statistical algorithm “Wey’s urn”. The two groups (95 subjects each) were provided with two different products as follows: one group used the probiotic plus multivitamin food supplement (active treatment), while the other group used the multivitamin food supplement only (control treatment). The active product contained two probiotic strains, named *Limosilactobacillus reuteri* PBS072 and *Bifidobacterium breve* BB077, for a final dosage of 4 × 10^9^ CFU/day (2 × 10^9^ CFU for each strain); a multivitamin mix (vitamins B1, B2, B3, B5, B6, B9, B12, D, and E); dried corn starch, magnesium stearate, and silicon dioxide. *L. reuteri PBS072 (DSM 25175)* and *B. breve BB077 (LMG P-30157)* were both isolated from the stool of a healthy human infant. The control treatment comprised the same multivitamin mix as the active product plus maltodextrin, magnesium stearate, and silicon dioxide. The products were administered in the form of capsules, and the subjects were instructed to take 1 capsule a day of the active/control product with a glass of water (not sparkling water) away from meals. The participants were encouraged to maintain their habitual daily routines. If antibiotics were prescribed, the women were encouraged to continue with their treatment regimen but advised to take the treatment at least 2 h after taking the antibiotic. Clinical visits were scheduled as follows: initial visit—recruitment (T-1), start of this study—first product intake (T0), intermediate visit (i.e., 45 days after the beginning of the treatment, T1), final visit (i.e., 90 days after the beginning of the treatment, T2). This study’s schedule is reported in Table 2. During the initial visit, the gynaecologist invited the subjects to communicate the date of delivery (corresponding to the beginning of product intake), in order to plan the date for the intermediate and final visits. This study’s timeline is illustrated in Figure 2.

### 2.2. Outcome Measures

The evaluation of depression-related symptoms was carried out by using the Edinburgh Postnatal Depression Scale (EPDS) [31]. The EPDS is a 10-item self-reporting questionnaire developed by Cox et al. to identify women who may suffer from postpartum depression [32]. The 10 questions of the scale correspond to various clinical depression symptoms, such as feeling guilty, sleep disturbance, low energy, anhedonia, and suicidal ideation. Each answer is given a score ranging from 0 to 3, with a maximum score of 30 points; a score of 10 or above suggests an intensification of the depressive state [33]. A score of 12 is interpreted as “fairly high possibility of depression”.

The evaluation of breastfeeding quality was performed using the Breastfeeding Self-Efficacy Scale—Short Form (BSES-SF) reported by Rashid et al. [34]. The BSES-SF is a 14-item instrument developed to measure breastfeeding confidence, based on a 5-point Likert-type scale, ranging from 1 (not at all confident) to 5 (always confident), with a maximum score of 70. The higher the score, the better the quality of breastfeeding. In addition to the 14 items of the BSES-SF, 3 more questions were added regarding crying/fussing and possible mastitis events. The mothers were asked to report, with a yes or no, whether they noted improvement. Furthermore, the subjects were invited to quantify the average daily number of baby crying episodes during the treatment.

The questionnaires were completed at T1 and T2. At T0, no questionnaire was filled since, generally, depression symptoms appear within 30–40 days from delivery [3]. 

### 2.3. Statistical Analysis

Statistical analysis was performed using NCSS 10 Statistical Software (version 10.0.7 for Windows; NCSS, Kaysville, UT, USA) running on Windows Server 2008 R2 Standard SP1 64-bit edition (Microsoft, Redmond, WA, USA). The data normality was checked using the Shapiro–Wilk W normality test and data shape. Intergroup (active vs. control) comparisons were carried out using the Mann–Whitney U test. The data were expressed as mean ± standard deviation (SD). A *p*-value < 0.05 was considered statistically significant. Statistical analysis was carried out on raw data. 

## 3. Results

### 3.1. Depression Symptom Evaluation

**Figure 3:**
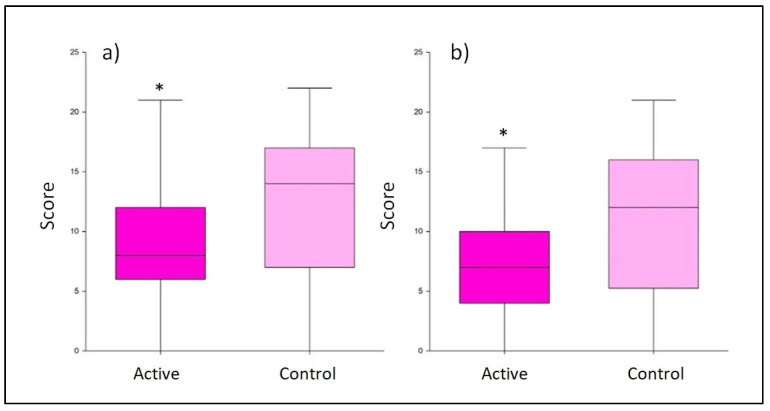
Score obtained from the Edinburgh Postnatal Depression Scale questionnaire. Comparison between the active and control products at T1 (**a**) and T2 (**b**). The boxplots show the minimum, the median, and the maximum of the values collected. Statistical differences were calculated using Mann–Whitney U test: * *p*-value < 0.001.

The evaluation of depression symptoms was conducted using the Edinburgh Postnatal Depression Scale, after 45 and 90 days of active/control treatment (T1 and T2, respectively). At T1, the active treatment group reached a total score that was underneath the limit of minor depression (i.e., 10 points) with an average score of 9.0 ± 4.8, while the control group reached a mean value of 12.1 ± 5.9 points. The result obtained for the active treatment was significantly lower than that for the control (*p* < 0.001; Figure 3). This difference was even more evident at T2. At this time point, the use of the probiotic treatment produced a significantly lower average score of 7.0 ± 3.3 compared to the control group, which scored a mean value of 10.8 ± 6.2 (*p* < 0.001; Figure 3).

### 3.2. Breastfeeding Quality Assessment

**Figure 4:**
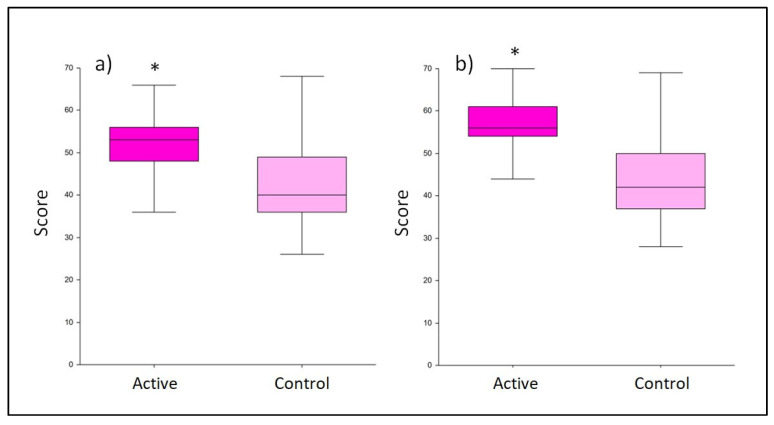
Score obtained from the Breastfeeding Self-Efficacy Scale—Short Form (BSES-SF) questionnaire. Comparison between active and control at (**a**) T1 and (**b**) T2. The boxplots show the minimum, the median, and the maximum of the values collected. Statistical differences were calculated using Mann–Whitney U test: * *p*-value < 0.001.

The breastfeeding quality was determined using the BSES-SF questionnaire filled by subjects after 45 and 90 days of treatment. The active product showed a significant improvement in the average score obtained with respect to control already at T1 (51.46 ± 9.1 vs. 42.80 ± 10.7, respectively, with *p* < 0.001; Figure 2). After 90 days of treatment, the probiotics seemed to still exert a positive, significant effect on the breastfeeding quality (*p* < 0.001), reaching an average value of 56.37 ± 7.9, while at the same time, the control average score was 45.48 ± 11.9 (Figure 4).

### 3.3. Baby’s Crying/Fussing Events

**Figure 5:**
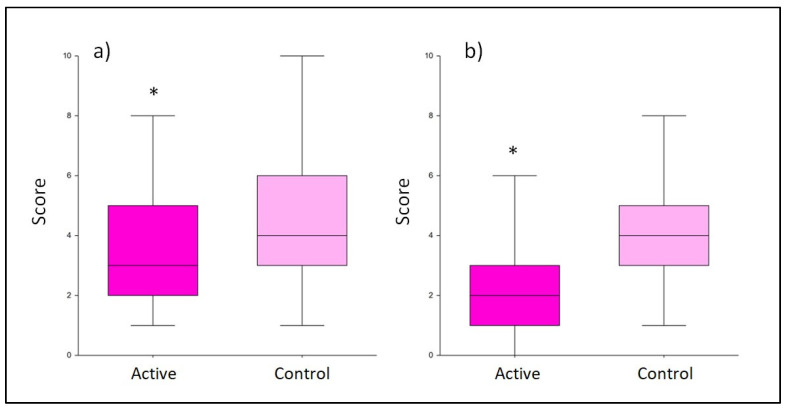
Average number of crying/fussing events. Comparison between the active and control product at T1 (**a**) and T2 (**b**). The boxplots show the minimum, the median, and the maximum of the collected values. Statistical differences were calculated using Mann–Whitney U test: * *p*-value < 0.001.

Regarding the daily improvement in crying/fussing events, a positive effect was observed in the group treated with probiotics. At T1, considering the positive answer to item 15 the of the BSES-SF questionnaire (assessing the crying/fussing events during the treatment), 81% of the active group reported an improvement in comparison to 42% in the control group (Table 3). The difference in the percentage of positive answers was confirmed at T2 also, where the active and control groups reached 78% and 43%, respectively. In addition, the average number of crying/fussing events was significantly lower (*p* < 0.001) in the probiotic-treated group with respect to the multivitamin product, both at T1 and T2 (Figure 5).

## 4. Discussion

According to the World Health Organization, stress has been defined as the “Health Epidemic of the 21st Century”. Indeed, the physical and psychological burden caused by long stress periods is increasing [35]. Stressful situations can affect the quality of sleep, provoking fatigue, irritability, and concentration difficulties. Moreover, stress may lead to intestinal dysbiosis and modify the GM and, consequently, the gut–brain communication. Therefore, keeping a balanced gut microbiota through probiotic supplementation could be a valid solution to achieve effective gut–brain crosstalk, resulting in an improvement in mental conditions, as reported by previous studies [22,36,37]. Studies on animal models of depression reveal that the GM of depressed mice differed significantly from that of healthy subjects. Indeed, the GM modulates neuroinflammatory activity in the hippocampus through a dysfunctional microbiota–gut–brain axis, leading to anxiety- and depression-like phenotypes [38]. Similarly, a study revealed that faecal microbiota transplantation (FMT) from human patients with major depressive disorder (MDD) into rats could induce a depressive-like phenotype in the recipient animals [39]. Rats receiving an FMT from depressed humans showed significantly higher immobility and less struggling in the forced swim test than rats receiving an FMT from healthy human donors, a behaviour interpreted as a depressive phenotype. Furthermore, the former group showed an altered intestinal tight junction gene expression compared to animals receiving an FTM from healthy donors [39]. These data highlight the key role of microbiota in modulating depression.

Nowadays, it is well known that probiotic supplementation in women during the last trimester of gestation and breastfeeding shapes newborns’ immune systems. According to the 2015 World Allergy Organization guidelines, pregnant women whose infants are at high risk for allergy should assume probiotics as a preventive treatment. Indeed, probiotic integration positively stimulates the children’s immune system, decreasing the onset of allergic diseases [40].

The majority of scientific studies available in the literature evaluate the immune system response and the GM composition of newborn babies in women taking probiotic supplements during the perinatal period [41]. However, the effects of probiotic supplementation on new mother’s mental health both during pregnancy and in the puerperium have been rarely assessed. Indeed, in the puerperium, mothers experience several physiological changes that could lead to stress and anxiety. Progesterone and oestrogen, two hormones that increase tenfold during pregnancy, dramatically drop after the delivery. This condition could persist for 3–4 weeks, and it is related to many changes in the mother’s life, such as diurnal variations in mood, poor concentration, and irritability [7]. Usually, this condition self-resolves without any intervention, but sometimes, these mental states could last longer, leading to more serious discomforts, such as PPD [5].

The aim of our study was to assess whether a supplement containing *Limosilactobacillus reuteri PBS072* and *Bifidobacterium breve BB077* plus multivitamins may provide a safe support to new mothers after delivery, especially improving their mental state. Indeed, we observed the effect on mood of two probiotic strains administered to mothers immediately after delivery, in order to prevent the onset of depressive and anxious symptoms linked to PPD development, together with the indirect influence of the probiotic intake on newborns during the first trimester. 

The evidence that women supplemented with probiotics had fewer symptoms of postnatal anxiety and depression caused by stress is consistent with two previous clinical studies evaluating the effect of probiotics on stress-related parameters in different target populations. The first study is a proof-of-concept trial of 30 students enrolled during an exam session and treated with a probiotic supplement containing the same strains *L. reuteri* PBS072 and *B. breve* BB077. The results of that study showed an improvement in the student’s cognitive function and sleep quality [29]. In the second study, Nobile et al. reported an improvement in the general mood state, anger, and tension feelings, as well as sleep quality, in employees administered the same probiotic blend during the pandemic lockdown, suggesting its potential role in the management of work-related stress [30]. 

According to this preliminary evidence, our study was designed to assess the effect of probiotics on mood and mental state in the period in which the first PPD symptoms emerge. It is worth noticing that the clinical protocol could not include evaluation questionnaires at T0 since they are not relevant before the occurrence of different and opposite emotional events (e.g., from excitement and joy to weakness and fright due to motherhood). However, this study demonstrated a significant improvement in mothers’ mood in the first trimester when supplemented with *Limosilactobacillus reuteri* PBS072, *Bifidobacterium breve* BB077, and vitamins compared to the control group according to the EPDS questionnaire (26% at T1 and 35% at T2, respectively). In addition, the probiotic group at T1 achieved a total score underneath the threshold of minor depression, while the control group reached a mean value of 12, which is interpreted as “fairly high possibility of depression” on the EPDS [32]. In the same way, this study demonstrated a significant improvement in breastfeeding quality in the probiotic group according to the BSES-SF (20% at T1 and 24% at T2, respectively). This questionnaire is associated with self-confidence during breastfeeding, assessed as lactation performance in new mothers.

Remarkably, this study showed a significant daily improvement in baby crying/fussing events (*p* < 0.001), according to the BSES-SF questionnaire at both T1 and T2 for the probiotic group. A possible explanation could be that probiotics may ameliorate the mothers’ mood, who, being less stressed and happier, may differently perceive the crying episodes of their children in terms of the episodes’ number and severity. Also, it could be speculated that more self-confident and calm mothers positively influence their baby’s behaviour. A further potential explanation of this outcome is that crying events could be possibly linked to a reduction in infant colic mediated by the indirect probiotic intake through breastfeeding [42]. Existing scientific evidence correlates changes in the GM with colicky infants; thus, the use of probiotics to counteract this disorder has rapidly increased in recent years, focusing on the reduction in crying time as a primary outcome [43,44,45]. It is well known that bacteria present in the breast milk are transferred from the mother’s intestine to the mammary gland and, consequently, from the mother’s milk to the infant’s gut [41]. In this context, a balanced microbiota in the mother’s gut could influence the growth of the child’s resident bacterial population, preventing intestinal gas production. Therefore, probiotic assumption might reduce infant colic episodes, decreasing crying events and improving the mother’s mood [46]. 

To the best of our knowledge, only one other published study assessed PPD neurological parameters as a result of the effect of probiotics in new mothers. This study investigated the effect of *Lactobacillus rhamnosus* HN001 on postpartum depression and anxiety symptoms using the EPDS and State–Trait Anxiety Inventory (STAI) questionnaires [36]. Slykerman and colleagues studied the oral intake of a probiotic supplement throughout the entire perinatal period, up to 6 months from delivery. The data of this study were collected retrospectively, after the occurrence of the events (up to 6 months later). Due to the latency between the effective event’s occurrence and its registration, this design could be a limitation of that study since mothers may have not remembered the events correctly throughout the long administration/observational period.

In our study, the questionnaires used for the assessment of the mothers’ mood state and breastfeeding confidence lacked a basal score, as the selected questionnaires only referred to the postnatal period; indeed, they could not be properly filled at T0 since the mothers had just undergone delivery, without experiencing the wide range of emotional events linked to motherhood yet. For this reason, no evaluation was performed at T0 in order to avoid any alteration of the EPDS and BSES-EF questionnaires.

As secondary outcome, mastitis incidence was taken into consideration to further characterize the lactation performance. However, since the two items of the BSES-SF questionnaire related to mastitis did not record any episodes, this outcome was not considered.

## 5. Conclusions

The mechanism by which probiotics modulate the mood is still to be clarified; however, growing evidence suggests that probiotics influence the gut–brain axis.

The aim of the present study was to further investigate the efficacy of the two probiotics strains in a transitory stress condition, i.e., the postpartum period. In this case, early PPD could be considered as a novel condition to explore, considering its high prevalence in developed countries. 

Our study provides evidence that the administration of a probiotic supplement containing *L. reuteri* PBS072 and *B. breve* BB077 could reduce the possibility of anxiety and stress in mothers after delivery and prevent the onset of PPD. To support these promising results, further studies are required to define the specific mechanism of action. As a future step of the research, the effect of the administration of these probiotic strains on postpartum mood can be assessed in women with perinatal sadness states, to test whether postpartum depression can be prevented by administering a probiotic. Also, it would be interesting to determine any potential change in the intestinal microbiota through stool analysis. 

## Figures and Tables

**Table 1:** Inclusion and exclusion criteria of this study.

Inclusion Criteria	Exclusion Criteria
Good general health condition	Subjects who do not meet the inclusion criteria
Women in the first trimester postpartum	Subjects considered as not adequate to participate in this study by the investigator
Aged between 18 and 50 years old (extremes included)	Subjects with known or suspected sensitization to one or more test formulation ingredients
Willingness to breastfeed *	Adult protected by law (under control or hospitalized in public or private institutions for reasons other than research or incarcerated)
Willingness to use probiotics and multivitamin food supplements consigned at the last visit before delivery	Subjects not able to communicate or cooperate with the investigator due to problems related to language, mental retardation, or impaired brain function
Willingness to fill up questionnaires	Subjects suffering from other psychiatric disorders such as schizophrenia, other psychotic disorders, bipolar disorder, or substance use disorder
Willingness to use only the products to be tested during the entire study period	Subjects with serious physical illnesses or mental disorders
Willingness not to use similar products that could interfere with the product to be tested	Subjects with significant risk of infanticide according to the investigator’s assessment
Willingness not to vary the normal daily routine (i.e., lifestyle, physical activity, etc.)	Subjects taking herbal remedies or psychotropic drugs intended for depression and taken within the last 2 weeks prior to baseline or during this study
Subjects aware of this study’s procedures and having signed an informed consent form	Subjects receiving counselling or psychological therapies at baseline or during this study

* If the woman after delivery was not able to breastfeed, she still remained in the panel since maternal breastfeeding was a secondary outcome.

**Table 2:** Study schedule.

Study Phases	Initial Visit Recruitment (T-1)	Start of the Product Intake (T0)	Intermediate Visit (T1)	Final Visit (T2)
**Signed informed consent**	X	-	-	-
**Subject eligibility**	X	-	X	X
**Clinical assessment—safety of use**	-	-	X	X
**Filling up questionnaires supported by the gynaecologist**	-	-	X	X
**Product distribution**	X	-	-	-
**Unused product collection**	-	-	-	X

**Table 3:** Positive and negative answers to question number 15 of the Breastfeeding Self-Efficacy Scale—Short Form (BSES-SF). The question was related to the improvement recorded in the crying/fussing events during the treatment.

Treatment	Answer at T1		Answer at T2	
	Yes	No	Yes	No
**Active product** **(probiotics plus multivitamins)**	77	18	74	21
**Control** **(multivitamins only)**	40	55	41	54

## Data Availability

The data presented in this study are available on request from the corresponding author.
